# Prenatal cannabinoid exposure and early language development

**DOI:** 10.3389/fped.2023.1290707

**Published:** 2023-11-22

**Authors:** Maria M. Talavera-Barber, Evlyn Morehead, Katherine Ziegler, Christine Hockett, Amy J. Elliott

**Affiliations:** ^1^ Avera Research Institute, Sioux Falls, SD, United States; ^2^ Department of Pediatrics, University of South Dakota Sanford School of Medicine, Sioux Falls, SD, United States; ^3^ Chicago Medical School, Rosalind Franklin University of Medicine and Science, North Chicago, IL, United States

**Keywords:** cannabis, marijuana, neurodevelopment, language, motor, cognition, Mullen scores

## Abstract

**Introduction:**

The effect of prenatal cannabis exposure (PCE) on childhood neurodevelopment remains poorly understood. There is a paucity of studies describing the neurodevelopment impact of PCE in infancy. The Mullen Scale of Early Learning (MSEL) is a cognitive screening tool that can be used from birth to 68 months and includes language and motor domains. Here we aim to explore the association between PCE during pregnancy and neurodevelopmental outcomes at 12 months of age.

**Methods:**

Participants were pregnant persons/infant pairs enrolled in The Safe Passage Study, a large prospective cohort study. Inclusion criteria included data available on PCE with associated MSEL scores at 12 months of age. Exposed participants were defined as early exposure (1st trimester only) or late exposure (2nd or 3rd trimester) and were randomly matched with unexposed participants. Multiple linear regression models were performed to test associations between prenatal cannabis exposure and the five Mullen subscales: gross motor, fine motor, expressive language, receptive language, and visual reception.

**Results:**

Sixty-nine exposed and 138 randomly matched unexposed infants were included in the analyses. Mothers of children with PCE were younger with the mean age 23.7 years for early exposure (*n* = 51) and 22.8 years for late exposure (*n* = 18). Maternal characteristics with prenatal cannabis use include a high-school education, American Indian or Alaska Native descent, lower socioeconomic status and co-use of tobacco. There were no gestational age or sex difference among the groups. Expressive (95% CI: 2.54–12.76; *p* = 0.0036,) and receptive language scores (95% CI: 0.39–8.72; *p* = 0.0322) were significantly increased between late-exposed infants compared to unexposed infants following adjustment for covariates. Gross motor scores (95% CI: 1.75–13; *p* = 0.0105) were also significantly increased for early-exposed infants with no difference in visual reception scores.

**Conclusion:**

Preclinical studies have shown abnormal brain connectivity in offspring exposed to cannabis affecting emotional regulation, hyperactivity, and language development. Results from this study link PCE to altered early language development within the first year of life. Exposed infants demonstrated increased expressive and receptive language scores at 12 months of age, which can translate to better performance in school. However, further research is needed to determine the implications of these results later in childhood.

## 1. Introduction

With cannabis legalization on a steady rise, the prevalence of prenatal cannabis use has increased drastically. The incidence of use by pregnant individuals has increased from 3.4% in 2002 to 7% in 2017 with a large spike coinciding with the COVID-19 pandemic to 25% (1–3). Reasons for use during pregnancy range from nausea, migraines, insomnia, pain, anxiety, and stress relief (1, 4). Exposure to cannabis prenatally may be associated with neurodevelopmental and cognitive abnormalities in the developing fetus. The cannabis metabolite, *Δ*^9^-tetrahydrocannabinol (THC), crosses the placenta (5, 6) and interferes with the endocannabinoid system, which is associated with neurodevelopment (7, 8). There is a growing body of evidence associating prenatal cannabis exposure (PCE) to adverse neonatal and perinatal outcomes (3), such as low birth weight, preterm birth, and fetal growth restriction (9), as well as long-term neurodevelopmental outcomes (10, 11). Based on concerns for impaired neurodevelopment, as well as maternal and fetal exposure to smoking, the American College of Obstetricians and Gynecologist (ACOG) recommends avoiding the use of cannabis in women who are pregnant or contemplating pregnancy (12).

The current research on PCE and child outcomes is inconsistent due to the lack of cross-study replication, limited exposure detail, the inability to account for polysubstance use, and limited high-quality longitudinal studies that if adequately powered and supported by appropriate psychological assessment tools could inform later cognitive outcomes (13). Recent data from an ongoing longitudinal study, the Adolescent Brain Cognitive Development (ABCD) study, has linked PCE to adverse middle and later childhood outcomes including: (i) psychopathology, (ii) sleep disorders, (iii) lower cognition, and (iv) structural brain abnormalities (14). However, there remains a paucity of clinical evidence demonstrating neurodevelopmental changes as early as 12 months of age. Moreover, the timing of when these associations begin to occur is unknown. The Mullen Scales of Early Learning (MSEL) is a standardized neurodevelopmental assessment tool used by clinicians and researchers to assess the developmental functioning in children as young as 2 days old to 68 months. Five domains of cognitive development are assessed: (i) visual reception (ii) expressive and receptive language, and (iii) gross and fine motor skills (15). Higher scores reflect comprehension at a higher developmental level with lower scores indicating areas for improvement. MSEL has been used to characterize neurodevelopmental deficits, track changes over time, and detect areas for intervention. There are no studies utilizing MSEL testing in early infancy to detect neurodevelopmental changes in those prenatally exposed infants at various time points in pregnancy.

In this study, we used data from a large prospective cohort study, the Safe Passage Study conducted by the Prenatal Alcohol and SIDS and Stillbirth (PASS) Network, of mother-infant dyads in the Northern Plains to examine the association between PCE and the timing of exposure and MSEL scores in children 12 months of age. We hypothesized that PCE during pregnancy was negatively associated with MSEL scores in those cannabis-exposed infants compared to those unexposed. We further hypothesized infants exposed later in pregnancy would have significantly lower MSEL scores than those only exposed early in development or unexposed during pregnancy.

## 2. Methods

### 2.1. Study population

**Figure 1:**
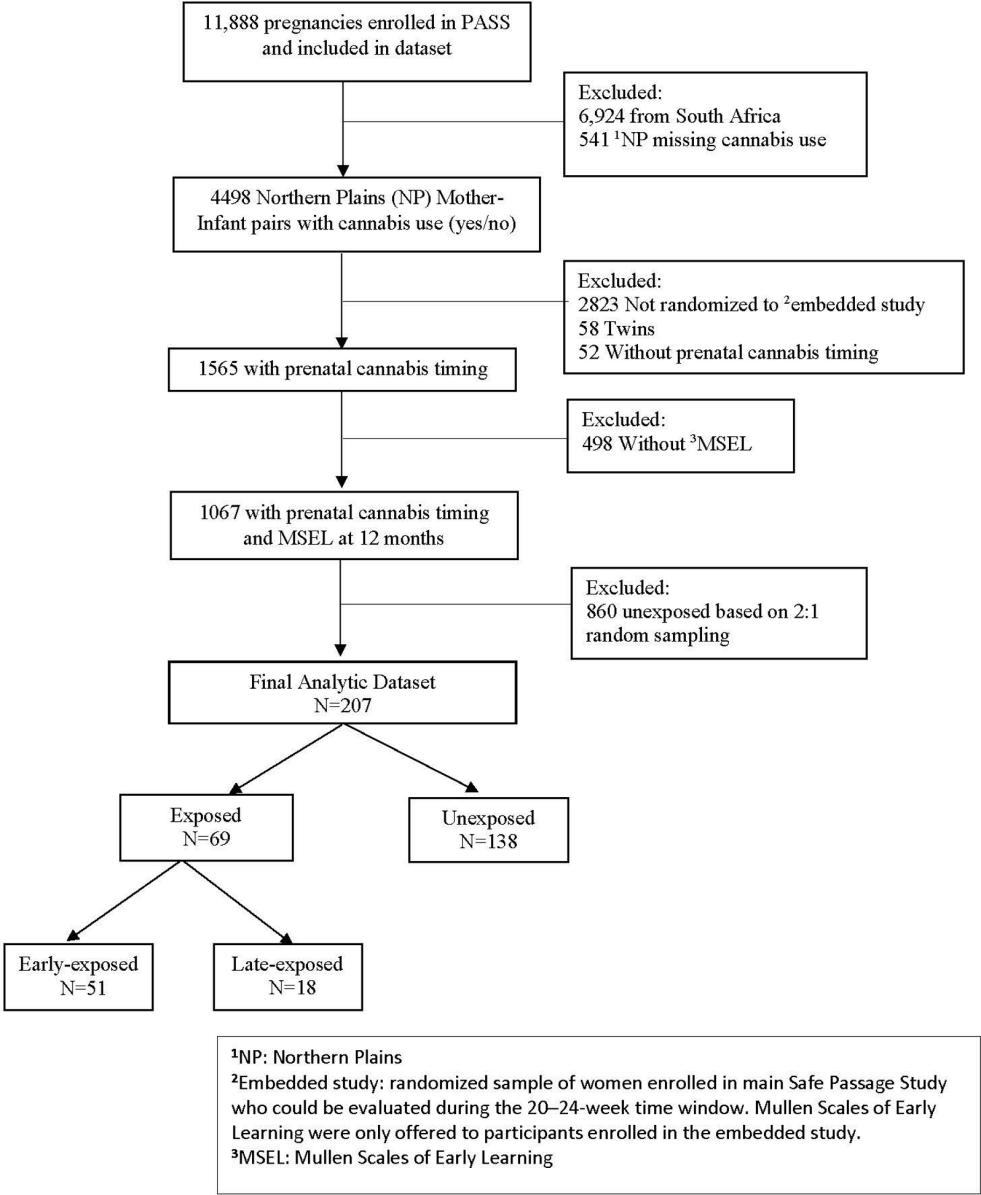
PASS study prenatal Cannabis Use CONSORT diagram. CONSORT format diagram describing study selection of participants exposed and unexposed to prenatal cannabis.

A subset of 207 pregnant individuals enrolled in the Safe Passage Study between August 2007 and January 2015 with postnatal follow-up through the first 12 months were included in this secondary analysis. The Safe Passage Study is a large, prospective study designed to investigate the association between prenatal exposures (primarily alcohol) to sudden infant death syndrome (SIDS), still birth, and determine biological basis for this increased risk. MSEL was collected from participants randomly selected to an embedded study within the larger Safe Passage Study. To be eligible for embedded study randomization women had to be enrolled in the main study and able to be evaluated at 20–24 weeks. Study design and methods have been previously described (16). Among the original 11,888 PASS pregnancies that provided substance use exposure data, we included those from the Northern Plains (5 sites in South Dakota and North Dakota) who responded to cannabis use questions, were randomized to the embedded study, and had an infant that completed the MSEL at the 12-month study visit. A random sampling (2:1, unexposed: exposed) was then performed within the PASS Northern Plains cohort to identify the unexposed group (Figure 1).

### 2.2. Data collection

#### 2.2.1. Prenatal cannabis exposure (PCE)

The primary exposure of prenatal cannabis use was collected via self-report. Participants were asked whether they used cannabis (referred to marijuana on data forms) and the date of last use at each of the three prenatal study visits. From this data, derived variables were created that identified cannabis use during pregnancy (yes/no) and cannabis use by trimester (yes/no). Trimester exposure was dichotomized into early (1st trimester) vs. late (2nd or 3rd trimester) based on the last date of use as reported by the participants. Therefore, late trimester exposure does include first trimester (throughout the whole pregnancy) exposure.

#### 2.2.2. Neurodevelopmental outcome

The primary outcome was child neurodevelopment at 12 months as measured by the MSEL. The MSEL was administered by trained Safe Passage study staff. The MSEL as a standardized neurodevelopmental assessment tool in 12-month-old infants based on its widely validated use in young infants with and without development and cognitive deficiencies (17–19). There are standardized separate subscales for gross motor, fine motor, visual reception, receptive language, and expressive language. These subscales have used in used in a variety of research analyses on diverse populations (20–22). The receptive language domain test auditory comprehension, auditory memory, and auditory sequencing. The expressive language domain tests speaking, language formation, and verbal conceptualization. The gross motor domain tests central motor and mobility. Higher scores on the MSEL test indicates that the child is demonstrating a skill level typical of an older child (23).The MSEL included five scales related to child neurodevelopment: (1) gross motor, (2) visual reception, (3) fine motor, (4) receptive language, and (5) expressive language and an optional early learning composite standard score. *T*-scores based on infant’s age and sex were calculated from summary raw scores based on child performance for each of the tasks within a scale.

#### 2.2.3. Covariates

Covariates of interest included demographics, such as age, race, education, monthly household income, and alcohol and tobacco use, gestational age (GA) in weeks at delivery, and infant sex. Prenatal anxiety was collected via the Spielberger State Trait Anxiety Index at the first prenatal visit. State anxiety is a measure of how one feels at the moment, and trait anxiety is a measure of how one generally feels. A score of >40 was defined as an indicator of anxiety. All covariates were collected via self-report. Alcohol use was collected using a modified 30-day Timeline Followback interview (16, 24).

### 2.3. Statistical analysis

Descriptive statistics, counts and percentages for categorical variables, means and standard deviations for continuous variables, were calculated for pregnant persons and infant characteristics. Chi Square, Fisher’s Exact, and ANOVAs were performed for tests of association as appropriate. Based on Shapiro-Wilks tests, normality assumptions were violated for MSEL, therefore, medians and ranges were provided and Kruskal Wallis tests were performed for bivariate analyses. Simple and multiple linear regression models were used to determine whether infants exposed to prenatal cannabis use were significantly associated with differences in MSEL scores compared to infants unexposed to prenatal cannabis. Post hoc tests were performed to determine whether there were differences in MSEL scores between infants exposed late in pregnancy compared to infants exposed early in pregnancy. Multiple linear regression models adjusted for the following covariates: pregnant person’s age, race, education, monthly household income, prenatal alcohol use, prenatal tobacco use, Spielberger state and trait anxiety, gestational age at delivery, and infant sex. All statistical analyses were generated using SAS software, Version 9.4 of the SAS System. Copyright © 2023 SAS Institute Inc., Cary, NC, USA.

## 3. Results

### 3.1. Analysis of maternal characteristics

Among the 207 mother-infant dyads included in the analysis, most pregnant people were white (58%), high school educated (81%), had a monthly household income above $1,500 dollars (54%), and were on average 26 years old. Fifty-nine percent reported prenatal alcohol exposure and 30% reported prenatal tobacco exposure. Infants were delivered on average at 39 weeks and 53% were male. Table 1 provides these descriptive statistics by PCE group with corresponding *p*-values. Significant differences were observed between PCE groups and all maternal characteristics except prenatal alcohol exposure. Pregnant people with prenatal cannabis use were younger, less likely to have a high school education, more likely to be American Indian, more likely to have a monthly household income of $1,500 or less, and less likely to smoke tobacco, and more likely to experience anxiety, than pregnant people with no prenatal cannabis use. No significant differences were observed between PCE groups and infant characteristics. Mullen scores were not significantly associated with PCE in bivariate analysis except for the expressive language subscale (*p* = 0.03).

**Table 1:** Descriptive summary of maternal and infant characteristics.

Maternal Characteristics	Total study sample	Unexposed to cannabis during pregnancy	Early exposure to cannabis during pregnancy	Late exposure to cannabis during pregnancy	*p*-value
Mother/infant dyads	207	138	51	18	
Maternal Age, mean (SD)	26.4 (5.2)	27.8 (4.7)	23.7 (5.0)	22.8 (5.3)	**<0** **.** **0001**
High school education, *n* (%)					
No	39 (18.8)	12 (8.7)	18 (35.3)	9 (50.0)	**<0**.**0001**
Yes	168 (81.2)	126 (91.3)	33 (64.7)	9 (50.0)	
Race^a^, *n* (%)					
American Indian or Alaska Native	76 (36.7)	27 (19.6)	36 (70.6)	13 (72.2)	**<0**.**0001**
White	119 (57.5)	105 (76.1)	11 (21.6)	3 (16.7)	
Other/Unknown	12 (5.8)	6 (4.3)	4 (7.8)	2 (11.1)	
Monthly household income^b^, *n* (%)					
≤$1500	89 (43.0)	37 (26.8)	39 (76.5)	13 (72.2)	**<0**.**0001**
>$1500	112 (54.1)	98 (71.0)	10 (19.6)	4 (22.2)	
Prenatal alcohol exposure^b^, *n* (%)					
No	82 (39.6)	62 (44.9)	14 (27.5)	6 (33.3)	0.07
Yes	123 (59.4)	74 (53.6)	37 (72.5)	12 (66.7)	
Prenatal tobacco exposure^b^, *n* (%)					
No	139 (67.2)	114 (82.6)	19 (37.3)	6 (33.3)	**<0**.**0001**
Yes	61 (29.5)	19 (13.8)	30 (58.8)	12 (66.7)	
Spielberger State Anxiety^a,b^, *n* (%)					
No	187 (90.8)	130 (94.2)	40 (80.0)	17 (94.4)	**0**.**02**
Yes	19 (9.2)	8 (5.8)	10 (20.0)	1 (5.6)	
Spielberger Trait Anxiety					
No	174 (84.5)	126 (91.3)	35 (70.0)	13 (72.2)	**0**.**0006**
Yes	32 (15.5)	12 (8.7)	15 (30.0)	5 (27.8)	
Infant Characteristics					
Gestational age in weeks	39.4 (1.5)	39.3 (1.6)	39.4 (1.4)	39.9 (0.8)	0.22
Sex					
Male	110 (53.1)	78 (56.5)	27 (52.9)	5 (27.8)	0.07
Female	97 (46.9)	60 (43.5)	24 (47.1)	13 (72.2)	
MSEL, median (range)^c^					
Early Composite Standard Score	99 (51–134)	98 (51–134)	100 (71–117)	104.5 (89–121)	0.22
Expressive Language *T*-Score	51 (21–78)	46 (21–78)	51 (25–67)	57 (37–62)	**<0**.**03**
Fine Motor *T*-Score	55 (20–74)	55 (20–74)	49 (20–69)	55 (28–65)	0.92
Gross Motor *T*-Score	53 (20–80)	53 (20–80)	57 (31–78)	55.5 (31–66)	0.25
Receptive Language *T*-Score	44 (22–73)	44 (22–73)	44.5 (35–64)	47 (34–60)	0.06
Visual Reception *T*-Score	52 (24–80)	52 (24–80)	47 (34–60)	52 (38–66)	0.19

Bolded values are statistically significant.

^a^ Fishers Exact test performed due to small cell size.

^b^ Percentages will not add up to 100% due to missing values;.

^c^ Kruskal Wallis test performed due to non-normal distributions.

### 3.2. Analysis of MSEL scores by trimester

Table 2 provides the results of unadjusted and adjusted linear regression models. Each model compared MSEL scores of PCE-infants by timing to unexposed infants. Post hoc tests were performed to compare MSE scores of late-exposed infants compared to early-exposed and to compare MSE scores of infants with any exposure to unexposed infants. In unadjusted models, significant associations were observed when comparing late-exposure to unexposed infants for the early composite standard (*p* = 0.04), expressive language (*p* < 0.004), and receptive language (*p* < 0.05) scores. A significant association was observed when comparing early-exposed only to unexposed infants for the gross motor score. A significant association was observed when comparing any exposure to unexposed infants for expressive language (*p* < 0.004) and receptive language (*p* < 0.02). These associations remained significant in multivariable linear regression models adjusting for maternal age, race, education, monthly household income, prenatal alcohol use, prenatal tobacco use, state and trait anxiety, gestational age at delivery, and infant sex (Table 2).

**Table 2:** Unadjusted and adjusted linear regression models of Mullen Scales of Early Learning (MSEL) by early and late exposure during pregnancy.

MSEL scores	Unadjusted				Adjusted^a^			
	Parameter estimate	95% Confidence Intervals		*p* value	Parameter estimate	95% Confidence Intervals		*p* value
		Lower	Upper			Lower	Upper	
Early composite standard score								
Any vs. None	2.89	−1.46	7.23	0.19	4.56	−1.20	10.32	0.12
Early vs. None	0.9	−4.11	5.91	0.72	2.40	−3.92	8.72	0.45
Late vs. None	**6** **.** **98**	**0**.**23**	**13**.**73**	**0**.**04**	**8**.**63**	**1**.**02**	**16**.**23**	**<0**.**03**
Late vs. Early Only	6.08	−1.62	13.78	0.12	6.23	−1.43	13.88	0.11
Expressive language *T*-Score								
Any vs. None	**3**.**99**	**1**.**25**	**6**.**75**	**<0**.**004**	**4**.**96**	**1**.**29**	**8**.**63**	**0**.**008**
Early vs. None	2.93	−0.19	6.06	0.07	3.81	−0.31	7.93	0.07
Late vs. None	**6**.**48**	**2**.**05**	**10**.**91**	**0**.**004**	**7**.**65**	**2**.**43**	**12**.**87**	**0**.**004**
Late vs. Early Only	3.55	−1.43	8.52	0.16	3.84	−1.34	9.02	0.15
Fine motor *T*-Score								
Any vs. None	0.34	−3.31	3.99	0.85	0.24	−4.86	5.35	0.93
Early vs. None	−0.35	−4.53	3.82	0.87	−0.70	−6.30	4.89	0.80
Late vs. None	1.92	−3.95	7.79	0.52	2.14	−4.72	9.01	0.54
Late vs. Early Only	2.27	−4.34	8.89	0.5	2.84	−4.02	9.71	0.41
Gross motor *T*-Score								
Any vs. None	4.00	0.08	7.93	0.05	**7**.**23**	**1**.**90**	**12**.**57**	**0**.**01**
Early vs. None	**4**.**75**	**0**.**28**	**9**.**21**	**<0**.**04**	**8**.**34**	**2**.**55**	**14**.**13**	**0**.**01**
Late vs. None	2.23	−4.17	8.62	0.49	4.75	−2.58	12.08	0.20
Late vs. Early Only	−2.52	−9.68	4.64	0.49	−3.59	−10.86	3.68	0.33
Receptive language *T*-Score								
Any vs. None	**2**.**68**	**0**.**46**	**4**.**90**	**<0**.**02**	**3**.**19**	**0**.**08**	**6**.**30**	**0**.**04**
Early vs. None	2.26	−0.27	4.79	<0.08	2.63	−0.76	6.02	0.13
Late vs. None	**3**.**67**	**0**.**08**	**7**.**26**	**<0**.**05**	**4**.**40**	**0**.**15**	**8**.**65**	**0**.**04**
Late vs. Early Only	1.41	−2.61	5.44	0.49	1.77	−2.46	6.00	0.41
Visual reception *T*-Score								
Any vs. None	−0.73	−3.81	2.34	0.64	2.70	−1.40	6.80	0.20
Early vs. None	−2.06	−5.54	1.42	0.24	1.56	−2.87	5.99	0.49
Late vs. None	2.43	−2.55	7.40	0.34	5.28	−0.34	10.89	0.07
Late vs. Early Only	4.49	−1.08	10.05	0.11	3.71	−1.83	9.26	0.18

Bolded values are statistically significant.

^a^ Pregnant person’s age, race, education, monthly household income, prenatal alcohol use, prenatal tobacco use, Spielberger state anxiety, Spielberger Trait anxiety, gestational age at delivery, and infant sex.

Among infants who were exposed late in pregnancy, we observed higher MSEL scores for early composite, expressive language, and receptive language when compared to unexposed infants after adjusting for covariates. Such that for infants who were exposed late in pregnancy, the predicted early composite standard score would be 8.63 points higher than infants unexposed to prenatal cannabis (95% CI:1.02–16.23; *p* < 0.03). The predicted expressive language t-score and receptive language t-score among infants who were exposed late in pregnancy would be 7.65 points (95% CI: 2.43–12.87; *p* = 0.004) and 4.40 points (95% CI: 0.15–8.65; *p* = 0.04) above unexposed infants. We observed a significant 8.34-point increase in predicted gross motor *t*-score only when comparing infants exposed early in pregnancy to unexposed infants (95% CI: 2.55–14.13; *p* = 0.01) after adjusting for covariates. There were no significant differences between exposure groups for fine motor and visual reception scales. No significant differences were observed in any MSEL scores in our *post hoc* comparisons between late exposure during pregnancy vs. early exposure during pregnancy. A significant 4.96-point increase in predicted expressive language *t*-score (95%CI: 1.29–8.63, *p* = 0.008) and a significant 3.19-point increase in receptive language *t*-score (95%CI: 0.08–6.30, *p* = 0.04) when comparing any prenatal cannabis exposure infants to infants with no exposure after adjusting for covariates.

## 4. Discussion

Epidemiological studies examining PCE and childhood cognition and development have been mixed with regard to the potential impact on the offspring. Although recent evidence has pointed to potentially deleterious effects of prenatal cannabis use, there is still mixed evidence as to the neurodevelopment effects across the life stages. The main objective of this study was to investigate the associations between PCE and neurodevelopmental domains in term infants around 12 months of life in a large, prospective cohort. Consistent with other research (2, 25), younger pregnant persons were more likely to use cannabis in pregnancy with lower socioeconomic status, more likely to experience anxiety, and co-use of tobacco during pregnancy. Our results demonstrated that infants exposed to cannabis later in pregnancy scored higher in overall early composite score, expressive, and receptive language compared to unexposed infants, and infants exposed to cannabis in early pregnancy scored higher in gross motor scores compared to unexposed infant. No differences were seen in fine motor or visual reception scores between the PCE groups.

We originally hypothesized that PCE would be associated with adverse cognitive (language, motor, or visual reception) outcomes for offspring exposed during pregnancy. We based this hypothesis on preclinical evidence suggesting that THC directly impacts fetal brain development. THC is a highly lipophilic molecule that readily crosses the placenta with the potential to accumulate in the fetal and placental tissues but has also been shown to have lower rates of deposition in fetal tissue, suggesting a role of the placenta in limiting THC exposure (6). THC can affect the placental transcriptome and the placental and fetal epigenome altering gene expression involved in neurobehavioral development (26, 27). Preclinical studies have described the critical role of the endogenous endocannabinoid system (ECS) in early brain development, particularly on neurogenesis, neuronal migration, axonal growth, and synaptogenesis, setting the stage for later in life behaviors (e.g., learning, memory) (7, 28–30). This suggests transient disruption of endogenous cannabinoid signaling from a variety of external or environmental stimuli can have a myriad of downstream effects depending on the timing and degree of exposure. However, we have limited data regarding exposures to suggest potential environmental or external stimuli that might have impacted downstream effects of PCE.

Cannabinoid receptor distribution changes over development with an increased number of cannabinoid receptors found in fetal and neonatal human brains (31). Cannabinoid receptors are widely distributed in the brain with highest densities in the hippocampus, cerebellum, and basal ganglia, and there is significant interaction with dopaminergic signaling, evidenced by associated euphoria and impact on cognition, memory, and attention (32, 33). Dopaminergic signaling has been linked to the development of speech initiation (34), vocalization (35), movement and cognition (36). Exogenous cannabinoids (THC) alter endogenous cannabinoid signaling by competing with endogenous cannabinoids and globally increasing cannabinoid signaling. Prenatal exposure to cannabinoids has been associated with changes in dopaminergic signaling with disruptions to the developmental trajectory of the dopaminergic system having capacity to produce both immediate and delayed effects. Based on this potential to change developmental trajectory, we can explain the improved 12 month MSLE scores with an unclear impact (positive or negative) anticipated at later timepoints.

Contrary to our hypothesis, prenatal exposure to cannabis was associated with significantly higher scores in gross motor, receptive and expressive language in infants following adjustment for covariates and stratification by trimester of exposure. Other epidemiological studies have demonstrated that PCE was associated with improved cognition scores (37), comprehension (38), and motor control (39, 40) in children ranging from 12 months to 12 years old. Animal studies have mixed evidence of locomotor and exploratory behavior with some studies demonstrating decreased locomotion and more time spent in immobility (41, 42) while others showed increased locomotor activity that ceased with removal of THC exposure (43) and showed sex-differences in the postnatal period (44). Interestingly, THC-exposed rat pups demonstrated increased hyperactivity, emotional reactivity, and anxiety in the form of increased ultrasonic vocalizations when separated from the dam (45). These cognitive differences were significantly different later in pregnancy (2nd or 3rd trimester) compared to those unexposed. A potential biological pathway that could explain these differences in trimester exposure is the CB1R pathway that is known to be highly concentrated in the human fetal mesocorticolimbic system, particularly the amygdala and hippocampus (46). Expression of CB1R is highest during mid-gestation then dropping precipitously after birth indicating mid-gestation may be the developmental period of highest susceptibility to THC (46). We also acknowledge that the impact of PCE is influenced by windows of increased vulnerability in the developing brain dependent upon stage of development (e.g., neurogenesis, synaptogenesis, presence of dopaminergic signaling/receptors).

A significant limitation to our study includes the lack of characterization of cannabis used including the frequency, mode, or quantity during the pregnancy limiting our ability to interpret the extent of drug exposure which could have ranged from minimal to consistent. However, our trimester stratification adds to the limited data on the timing of cannabis exposure during pregnancy. Maternal factors such as variation in placental transport, frequency/quantity used, and other potential unmeasured confounders could have a larger impact on smaller late-exposed infants (*n* = 18) group compared to early or unexposed infants. Despite biological evidence supporting the potential for negative phenotypes in those infants exposed to prenatal cannabinoids, the clinical evidence is mixed allowing for the potential of prenatal and postnatal environmental factors opportunity to mitigate negative outcomes. Other limitations of our study include the self-report methodology and lack of information on the extent of postnatal exposure to the infant with regard to second-hand exposure or via breastfeeding practices. A recent study by Moore et al. determined that postnatal exposure to cannabis was associated with more aggressive behavior, attention deficit/hyperactivity problems, oppositional/defiant behaviors and less cognitive flexibility in 5 year old children. Whereas, PCE was associated with fewer internalizing behaviors and fewer somatic complaints (47). In addition, we have examined one particular point in time (12 months) and have not adjusted for multiple comparisons such that interpretation of the data must be done within this context. The strengths of our study include a large, ethnically diverse, cohort with trimester specific-exposure and specialized neurodevelopmental data. In addition, the prospective, observational nature of the study allowed for a reduction of recall bias. However, we acknowledge the possibility of selection bias for those mothers with PCE that would be willing/able to participate in a longitudinal study could also be more invested in improving environmental factors postnatally such that these factors were not accounted for in the study. The inclusion of socioenvironmental factors and adjustment for co-exposure to alcohol and tobacco use strengthened the validity of the results. We account for the two most common co-used drugs (alcohol and tobacco) in this study but are limited in reporting exposure to other illicit substances include prescribed drugs. The limitations of this study, primarily the lack of known postnatal exposure, dampens the implication that PCE improves early language development and should be interpreted carefully within the context of the study.

In conclusion, our study indicated that the effects of PCE on neurodevelopment outcomes are established in infancy, and more specifically that the timing of exposure impacts development. More research is needed to better understand the impact of dose and frequency of cannabis use, as well as, understand the long-term effects over the life course. This is important to clinical healthcare and public health as a growing number of states are legalizing marijuana for medicinal and recreational purposes.

## Data Availability

The data analyzed in this study is subject to the following licenses/restrictions: Data sharing is restricted due to tribal restrictions. Requests to access these datasets should be directed to Amy.Elliott@avera.org.
